# Adventitial gene transfer of catalase attenuates angiotensin II-induced vascular remodeling

**DOI:** 10.3892/mmr.2014.3069

**Published:** 2014-12-10

**Authors:** CUN-FEI LIU, JIA ZHANG, KAI SHEN, PING-JIN GAO, HAI-YA WANG, XIN JIN, CHAO MENG, NING-YUAN FANG

**Affiliations:** 1 Department of Geriatrics, Renji Hospital, Shanghai Jiao Tong University School of Medicine, Shanghai 200001, P.R. China; 2 Division of Cardiology, Zhoushan People’s Hospital, Zhoushan, Zhejiang 316000, P.R. China; 3 Shanghai Key Laboratory of Vascular Biology at Ruijin Hospital and Shanghai Institute of Hypertension, Shanghai Jiao Tong University School of Medicine, Shanghai 200025, P.R. China; 4 Laboratory of Vascular Biology, Institute of Health Science Center, Shanghai Institute for Biological Sciences, Chinese Academy of Sciences, Shanghai 200025, P.R. China

**Keywords:** gene transfer, catalase, angiotensin II, vascular remodeling

## Abstract

Vascular adventitia and adventitia-derived reactive oxygen species (ROS) contribute to vascular remodeling following vascular injury. A previous *ex vivo* study in adventitial fibroblasts showed that catalase, one of most important anti-oxide enzymes, was downregulated by angiotensin II (AngII). The aim of the present study was to investigate whether adventitial gene transfer of catalase affects AngII-induced vascular remodeling *in vivo*. Adenoviruses co-expressing catalase and enhanced green fluorescent protein (eGFP) or expressing eGFP only were applied to the adventitial surface of common carotid arteries of Sprague-Dawley rats. Alzet minipumps administering AngII (0.75 mg/kg/day) were then implanted subcutaneously for 14 days. Systolic blood pressure and biological parameters of vascular remodeling were measured in each group. Adventitial fibroblasts were cultured and p38 mitogen-activated protein kinase (MAPK) phosphorylation was measured using western blot analysis. The results showed that adventitial gene transfer of catalase had no effect on AngII-induced systolic blood pressure elevation. However, catalase adenovirus transfection significantly inhibited AngII-induced media hypertrophy compared with that of the control virus (P<0.05). In addition, catalase transfection significantly attenuated AngII-induced ROS generation, macrophage infiltration, collagen deposition and adventitial α-smooth muscle actin expression. Furthermore, catalase transfection significantly inhibited the AngII-induced increase in p38MAPK phosphorylation. In conclusion, the results of the present study demonstrated that adventitial gene transfer of catalase significantly attenuated AngII-induced vascular remodeling in rats via inhibition of adventitial p38MAPK phosphorylation.

## Introduction

Reactive oxygen species (ROS) are known to have an important role in atherosclerosis and associated vascular diseases (1–3). In vascular physiology and pathophysiology, the majority of previous studies have focused on the role of the endothelium and vascular smooth muscle cells (VSMC) in ROS production (4,5). However, increasing evidence has suggested that vascular adventitia also had an important role in the process of vascular remodeling (6,7). Studies have shown that adventitia, rather than the intima or media, was the main source of ROS in angiotensin II (AngII)-induced hypertension (8); in addition, adventitial fibroblasts were reported to participate in AngII-induced vascular wall inflammation and remodeling (9). ROS production is primarily mediated via the activation of nicotinamide adenine dinucleotide phosphate (NADPH) oxidase in adventitia (10). A previous *ex vivo* study in adventitial fibroblasts demonstrated that AngII reduced the expression and activity of catalase, an important anti-oxidase, in a time- and dose-dependent manner (11). These studies therefore suggested that the imbalance between the pro-and antioxidant systems in adventitia may contribute to vascular remodeling following vascular injury.

Perivascular gene transfer of an adenoviral vector suspended in pluronic gel was found to be an efficient approach for the treatment of vascular injuries, which resulted in a minimal inflammatory response (12,13). Previous studies have shown that gene transfer of NADPH oxidase inhibitors into adventitia significantly attenuated vascular remodeling (8,14); however, there are a limited number studies which have investigated the role of anti-oxidative enzymes in the adventitia *in vivo* (15). In the present study, gene transfer techniques were used to determine whether overexpression of catalase through adventitial transfection improved AngII-induced vascular remodeling *in vivo*. The present study also investigated whether the p38 mitogen-activated protein kinase (MAPK) pathway was involved in the underlying mechanisms of AngII-induced vascular remodeling.

## Materials and methods

### Adenovirus (Ad) vector constructs

An adenoviral expression vector, Ad-cytomegalovirus-human catalase (hCAT)-internal ribosome entry site (IRES)-enhanced green fluorescent protein (eGFP) (Ad-CAT-eGFP), was constructed in order to co-express human catalase and eGFP, using the pAdEasy system (Stratagene, La Jolla, CA, USA). Therefore, eGFP expression indicated the expression of catalase. In brief, human catalase complementary DNA was cloned into the pShuttle-IRES-eGFP vector. The resultant shuttle plasmid was transformed into the *Escherichia coli* strain BJ5183 (Biochemistry Laboratory, Shanghai Jiao Tong University School of Medicine, Shanghai, China) containing the adenoviral backbone plasmid pAdEasy-1. Recombinant adenoviral plasmids were selected based on kanamycin (Sangon Biotech Inc., Shanghai, China) resistance and confirmed using restriction digestion. Virus particles were obtained through transfection of recombinant adenoviral plasmids into AD-293 cells (Invitrogen Life Technologies, Carlsbad, CA, USA). The virus was purified using CsCl banding (Amresco LLC, Solon, OH, USA) and the titer of the virus stock was determined using a plaque assay, as previously described (16). A control adenovirus Ad-eGFP which expressed eGFP only was also constructed.

### Animals and adventitial gene transfer of Ad-CAT-eGFP or Ad-eGFP

A total of 30 male Sprague-Dawley (SD) rats (6–8-weeks-old; 150–200 g) were purchased from the Shanghai Laboratory Animal Center of the Chinese Academy of Sciences (Shanghai, China) and maintained under environmentally controlled conditions (temperature, 20±2°C; 12-h light/dark cycle). Rats were anesthetized by intraperitoneal administration of sodium pentobarbital (30 mg/kg; Sigma-Aldrich, St. Louis, MO, USA), then the left common carotid artery was exposed (n=4–6 per group). Ad-CAT-eGFP or Ad-eGFP suspended in 200 μl pluronic F127 gel (20% wt/vol; Sigma-Aldrich) with a concentration of 1×10^9^ plaque forming units/ml, was carefully applied to the adventitial surface of the left common carotid artery (CCA). The incision was closed and the rats were allowed to recover from the anesthesia following coagulation of the gel. All protocols in the present study were approved by the Institutional Animal Care and Use Committee of Renji Hospital (Shanghai, China) and were consistent with the Guide for the Care and Use of Laboratory Animals (National Institutes of Health, Bethesda, MA, USA).

### AngII treatment and systolic blood pressure measurements

Two days post-adenovirus transfer, rats were anesthetized, as described above, and osmotic minipumps (2002; Alzet Osmotic Pumps, Cupertino, CA, USA) filled with AngII (Sigma-Aldrich) were implanted subcutaneously. The rate of AngII infusion was 0.75 mg/kg/day. Systolic blood pressure was measured using a computerized tail-cuff system (BP98A; Softron, Tokyo, Japan). Following 14 days of treatment, all 30 rats were euthanized by intraperitoneal injection of sodium pentobarbital (50 mg/kg) and CCAs were perfused with saline and then excised. The middle portions of vessels were fixed using 4% paraformaldehyde (Sigma-Aldrich) and embedded in paraffin (Sangon Biotech, Inc.).

### Morphometric and immunohistochemical analyses

Vessel segments were serially sectioned (5 μm) and then stained with modified hematoxylin and eosin (HE; Beyotime Institute of Biotechnology, Jiangsu, China). Cross-sectional images were captured using a light microscope (AxioSkop 20; Zeiss, Oberkochen, Germany). The thickness and area of the media and lumen were measured using Image-pro Plus 6.0 Software (Media Cybernetics, Inc., Rockville, MD, USA). The collagen content was measured using Picrosirius red staining (Beyotime Institute of Biotechnology), as previously described (17). A color threshold mask was defined in order to detect the red color by sampling. A negative background (black) was selected for thresholding and the positive area was measured by subtraction. The area of positive staining was recorded for each section. Results were obtained from two separate sections from 4–6 individual rats in each group. For immunohistochemistry, sections were deparaffinizated using xylene (Sangon Biotech, Inc.). and then rehydrated through a graded ethanol series (Beyotime Institute of Biotechnology), incubated with 0.3% hydrogen peroxide (Beyotime Institute of Biotechnology) for 20 min and then blocked with 5% goat serum (Beyotime Institute of Biotechnology), followed by incubation with the primary antibodies against eGFP (rabbit polyclonal anti-eGFP; 1:50; Cell Signaling Technologies, Danvers, MA, USA), α-smooth muscle actin (mouse monoclonal anti-α-SMA; 1:100; Sigma-Aldrich), 4-hydroxy-2-nonenal (rabbit polyclonal anti-4-HNE; 1:100; Abcam, Cambridge, MA, USA), CD68 (mouse monoclonal anti-CD68; 1:100; AbD Serotec, Kidlington, UK) and phosphorylated p38MAPK (rabbit polyclonal anti-phospho-p38MAPK; 1:100; Cell Signaling Technologies), followed by incubation with horseradish peroxidase-conjugated polyclonal goat anti-rabbit or goat anti-mouse secondary antibodies (1:200; Santa Cruz Biotechnology, Inc., Dallas, TX, USA). The reaction was visualized using diaminobenzidine and sections were counterstained with hematoxylin. Then the mean optical density (MOD) in each group was measured using Image-pro Plus Software.

### Cell culture

Adventitial fibroblasts (AFs) were isolated from thoracic aortas of male SD rats as previously described (9). Cells were grown in Dulbecco’s modified Eagle’s medium (DMEM; Invitrogen Life Technologies) containing 20% fetal bovine serum (FBS; Invitrogen Life Technolgies), 100 U/ml penicillin and 100 μg/ml streptomycin (Sangon Biotech, Inc.). Subconfluent cells were made quiescent by placing in 0% FBS-DMEM for an additional 24 h prior to intervention. AFs were then administered AngII (1×10^−7^ mol/l) or polyethylene glycol-catalase (PEG-catalase; 1,000 U/ml; Beyotime Institute of Biotechnology) in order to evaluate p38MAPK phosphorylation levels under different conditions.

### Western blot analysis

Following administration with different stimulants, total protein was extracted from cells using radioimmunoprecipitation assay lysis buffer in the presence of PMSF (Beyotime Institute of Biotechnology) and protein concentrations were measured using a bicinchoninic acid protein assay (Beyotime Institute of Biotechnology). Protein samples were separated using 10% SDS-PAGE and transferred to nitrocellulose membranes (Millipore, Bellerica, MA, USA). Following blocking with 5% non-fat milk or 5% bovine serum albumin, membranes were then incubated with primary antibodies against phosphorylated p38MAPK (rabbit monoclonal anti-phospho-p38MAPK; 1:1,000; Cell Signaling Technologies) and total p38MAPK (rabbit monoclonal anti-p38MAPK; 1:1,000; Cell Signaling Technologies), overnight at 4°C. The membranes were then washed and incubated with horseradish peroxidase-conjugated polyclonal goat anti-rabbit IgG secondary antibodies (1:2,000; Santa Cruz Biotechnology, Inc.) for 1 h. Western blots were developed using an enhanced chemiluminescence detection kit (PerkinElmer, Inc., Waltham, MA, USA) and quantified by densitometry using Gel-Pro Analyzer software 4.0 (Media Cybernetics, Inc.).

### Statistical analysis

Values are expressed as the mean ± standard deviation. Student’s t-test or one-way analysis of variance were performed in order to compare differences between two or multiple groups, respectively. P<0.05 and P<0.01 were considered to indicate a statistically significant difference between values. All statistical analyses were performed using SPSS software 16.0 (SPSS Inc., Chicago, IL, USA).

## Results

### Detection of adenovirus expression in CCA in vivo

**Figure 1:**
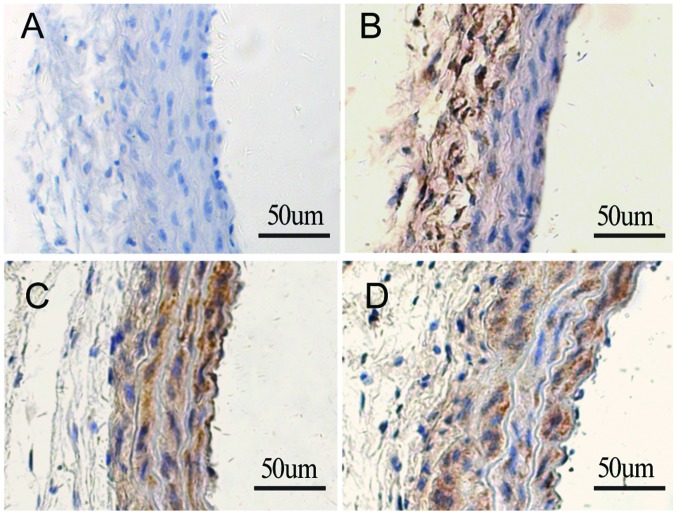
Detection of adenovirus expression in left common carotid arteries *in vivo* using immunohistochemistry with anti-eGFP. (A) Negative control, (B) Ad-CAT-eGFP transfection without AngII infusion, (C) AngII+Ad-CAT-eGFP and (D) AngII+Ad-eGFP (magnification, ×200; n=4–6). Ad, adenovirus; eGFP, enhanced green fluorescent protein; CAT, catalase; AngII, angiotensin II; CAT, catalase.

Immunohistochemical staining using antibodies specific to eGFP was performed to determine whether Ad-CAT-eGFP was successfully transfected into CCA following adventitial delivery. As shown in Fig. 1, the carotid arteries without gene transfer showed negative HE staining 14 days following minipump implant (Fig. 1A). In carotid arteries treated with Ad-CAT-eGFP without AngII infusion, positive staining was detected only in the adventitial layer (Fig. 1B). However, the carotid arteries of rats transfected with Ad-CAT-eGFP or Ad-eGFP with AngII infusion stained positively with HE, predominantly in media and neointima (Fig. 1C and D); this therefore indicated that adventitial cells migrated towards the media and neointima following vascular injury, as previously described (18).

### Catalase transfection has not effect on the hypertensive response to AngII

**Figure 2:**
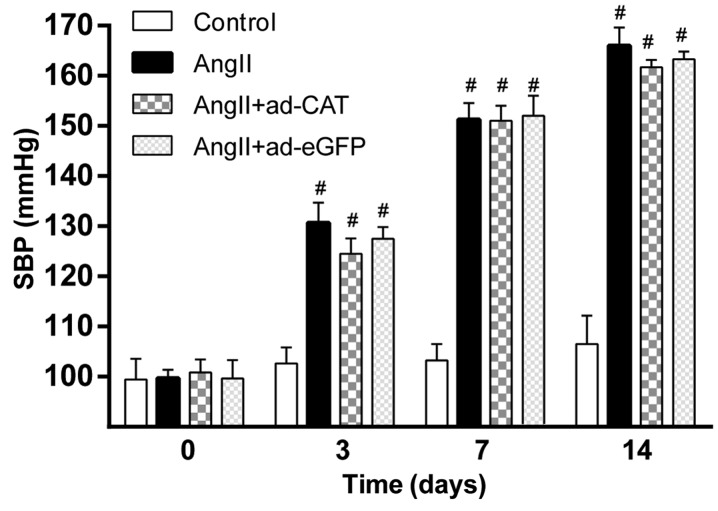
SBP 0, 3, 7 and 14 days following AngII infusion. Values are presented as the mean ± standard deviation (n=4–6). ^*^P<0.05 and ^#^P<0.01 vs. control. SBP, systolic blood pressure; AngII, angiotensin II; ad, adenovirus; eGFP, enhanced green fluorescent protein; CAT, catalase.

In order to determine the effect of Ad-CAT-eGFP transfection on blood pressure, the systolic blood pressure in each group was measured prior to and following AngII infusion (Fig. 2). Baseline systolic blood pressure was comparable among each experimental group at each time-point (P>0.05). In addition, systolic blood pressure was significantly increased in rats at 3, 7 and 14 days following AngII treatment compared to that of the control group (P<0.01); however, no significant differences were observed among the AngII, AngII+Ad-eGFP and AngII+Ad-CAT-eGFP groups. This therefore suggested that perivascular gene transfer of catalase has no obvious impact on systolic blood pressure.

### Catalase transfection ameliorates AngII-induced vascular hypertrophy

**Figure 3:**
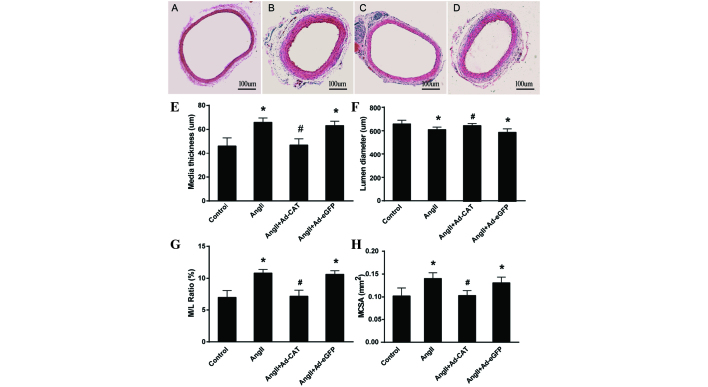
Ad-CAT-eGFP transfection attenuates AngII-induced characteristics of vascular hypertrophy. Immunohistochemistry images of section of the left common carotid artery in each group as follows: (A) Control, (B) AngII, (C) AngII+Ad-CAT-eGFP and (D) AngII+Ad-eGFP. Quantitative analysis of vascular hypertrophy characteristics, including (E) media thickness, (F) lumen diameter, (G) M/L ratio and (H) MCSA. Values are presented as the mean ± standard deviation (n=4–6). ^*^P<0.05 vs. control and ^#^P<0.05 vs. AngII or AngII+Ad-eGFP. eGFP, enhanced green fluorescent protein; CAT, catalase; ad, adenovirus; AngII, angiotensin II; M/L ratio, media to lumen ratio; MCSA, media cross-sectional area.

In order to determine whether the adventitial gene transfer of catalase attenuated AngII-induced vascular hypertrophy, the media thickness, media cross-sectional area (MCSA) and lumen diameter of the CAA of rats were measured in each group; comparisons were performed using sections taken from approximately the same area of the CCA. As shown in Fig. 3, AngII treatment significantly increased the media thickness, media-to-lumen ratio (M/L) and MCSA of the CCA compared to those of the untreated control, whereas the lumen diameter following AngII treatment was found to be decreased. In addition, transfection of Ad-CAT-eGFP significantly attenuated AngII-induced vascular hypertrophy (Fig. 3C); however, transfection of the Ad-eGFP vector did not attenuate vascular hypertrophy (Fig. 3D).

### Catalase transfection reduces α-SMA expression, ROS generation, macrophage infiltration and collagen deposition in the CCA

**Figure 4:**
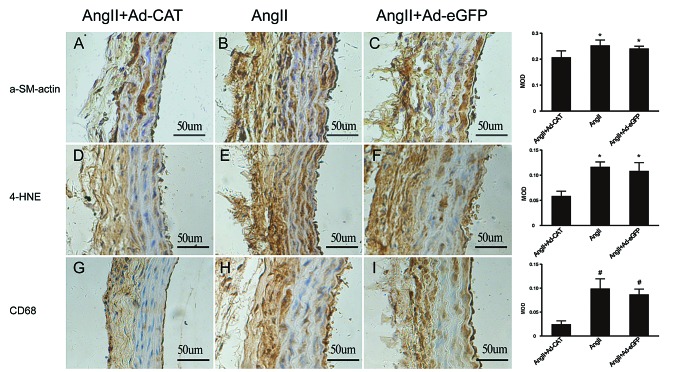
Catalase transfection attenuates AngII-induced vascular remodeling. Arteries were excised at day 14 following AngII infusion. Immunohistochemical analysis was performed to determine expression levels of (A–C) α-SMA, (D–F) 4-HNE and (G–I) CD68 in each AngII-treated group following control or catalase transfection. Bar graphs in the panel on the right show the quantified data as analyzed using a one-way analysis of variance to compare the MOD for each group. Values are presented as the mean ± standard deviation (n=4–6; magnification, ×200). ^*^P<0.05 and ^#^P<0.01 vs. AngII+Ad-CAT. AngII, angiotensin II; eGFP, enhanced green fluorescent protein; ad, adenovirus; CAT, catalase; α-SMA, α-smooth muscle actin; 4-HNE, 4-hydroxy-2-nonenal; MOD, mean optical density.

**Figure 5:**
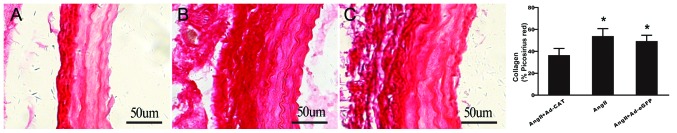
Collagen contents of the left common carotid arteries in each group were determined using picrosirius red staining. Collagen content following treatment with (A) AngII+Ad-CAT-eGFP, (B) AngII only and (C) AngII+Ad-eGFP (magnification, ×200). The bar graphs in the panel on the right show the quantified data. Values are presented as the mean ± standard deviation (n=4–6). ^*^P<0.05 vs. AngII+Ad-CAT. eGFP, enhanced green fluorescent protein; CAT, catalase; ad, adenovirus; AngII, angiotensin II.

In order to investigate whether catalase transfer improved vascular remodeling, measurements were taken of the following common markers of vascular remodeling using immunohistochemistry: α-SMA; 4-HNE, a lipid peroxidation by-product which reflects ROS generation; and CD68, which reflects macrophage infiltration. As shown in Fig. 4, adventitial transfection of Ad-CAT-eGFP significantly attenuated the expression levels of all of the above markers compared with those of the AngII and AngII+Ad-eGFP groups. Picrosirius red staining revealed that catalase overexpression markedly decreased collagen deposition compared with that in arteries treated with AngII only and AngII+Ad-eGFP (Fig. 5). These results demonstrated that catalase transfection significantly attenuated AngII-induced vascular remodeling.

### Catalase transfection attenuates AngII-induced vascular remodeling via inhibition of adventitial p38MAPK phosphorylation

**Figure 6:**
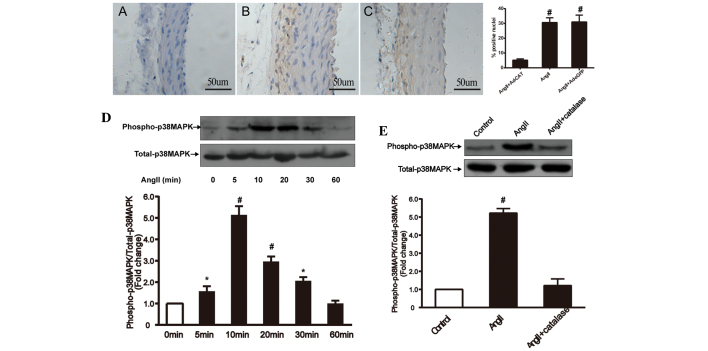
Catalase transfection inhibits AngII-induced phospho-p38MAPK expression in left common carotid arteries and adventitial fibroblasts *in vivo* and *ex vivo, respectively*. Immunohistochemical analysis of arteries treated with (A) AngII+Ad-CAT-eGFP, (B) AngII only and (C) AngII+Ad-eGFP (magnification, ×200). The bar graph in the panel on the right shows the percentage of hematoxylin-positive nuclei in each group (n=4–6). *Ex vivo* western blot analysis and quantification of (D) p38MAPK phosphorylation at 0, 5, 10, 20, 30 and 60 min following AngII treatment (1×10^−7^ mol/l) and (E) p38MAPK phosphorylation following pretreatment with polyethylene glycol-catalase (1,000 U/ml) for 30 min prior to exposure to AngII (1×10^−7^ mol/l) for 10 min in adventitial fibroblasts. Values are presented as the mean ± standard deviation (each experiment was conducted 6–8 times). ^*^P<0.05 vs. control and ^#^P<0.01 vs. control or AngII+catalase. AngII, angiotensin II; MAPK, mitogen-activated protein kinase; Ad, adenovirus; eGFP, enhanced green fluorescent protein; CAT, catalase.

p38MAPK is a potential critical component of the redox-sensitive signaling pathway, which is activated by AngII and ROS in numerous types of tissue and cells (19,20). In order to determine whether adventitial p38MAPK was involved in AngII-induced vascular remodeling and whether catalase attenuated AngII-induced vascular remodeling via inhibition of adventitial p38MAPK, immunohistochemical and western blot analyses were used to examine p38MAPK phosphorylation levels *in vivo* and *ex vivo* (Fig. 6). In the *in vivo* experiment, AngII infusion was found to significantly increase phospho-p38MAPK expression in the vascular adventitia, whereas following catalase transfection, adventitial phospho-p38MAPK expression was significantly decreased. In the *ex vivo* experiment, AFs were cultured and AngII (1×10^−7^ mol/l) or PEG-catalase (1,000 U/ml) was administrated in order to evaluate p38MAPK phosphorylation levels under different conditions. Western blot analysis revealed that AngII stimulation increased phospho-p38MAPK expression in AFs, which peaked at 10 min and then decreased again in a time-dependent manner. In addition, PEG-catalase treatment was shown to significantly inhibit AngII-induced phospho-38MAPK expression. These results therefore suggested that catalase overexpression attenuated AngII-induced vascular remodeling via inhibition of adventitial p38MAPK phosphorylation.

## Discussion

A previous *ex vivo* study demonstrated that adventitial catalase was downregulated by AngII in a time- and dose-dependent manner (11). The results of the present study showed that adventitial gene transfer of catalase into carotid arteries markedly attenuated characteristics of AngII-induced vascular remodeling, including α-SMA expression, ROS generation, macrophage infiltration and collagen deposition. In addition, the protective effects of catalase were found to be independent on blood pressure alterations. Furthermore, the present study indicated that the attenuation of AngII-induced vascular remodeling by catalase was, at least in part, mediated via inhibition of adventitia-derived p38MAPK phosphorylation.

Vascular adventitia is traditionally considered to provide support to the arterial wall; however, an increasing number of studies have suggested that adventitia may also have an important role in the regulation of vessel structure and function following exposure to a pathophysiological environment (21), including injury, hypoxia and hypertension (22,23). AFs are an important source of ROS in a variety of pathological conditions, such as AngII-induced hypertension (24); in addition, studies have shown that ROS generation was significantly increased in adventitia compared to that of the media or intima, when stimulated by AngII (8,25). Fibroblast-derived ROS, particularly H_2_O_2_ due to its stable and permeable nature, serve as intercellular signaling messengers, which may induce the development of vascular remodeling, leading to hypertension and atherosclerosis. In addition, when stimulated with AngII, AFs synthesize and release a variety of cytokines, including platelet-derived growth factor (26), interleukin 6 and monocyte chemotactic protein 1, which recruit macrophages and further promote vascular dysfunction (27). These studies therefore indicated the importance of adventitia in the process of AngII-induced vascular remodeling.

Adenovirus-mediated arterial gene transfection is a promising approach for the study of vascular biology and vascular gene treatment. Gene transfection into vascular adventitia was demonstrated to be effective and resulted in a minimal inflammatory response (12). Several studies have revealed that adventitial gene transfection into adventitial gene targets significantly ameliorated vascular remodeling under various conditions (8,14,28). However, the majority of studies to date have focused on inhibiting components of the pro-oxidant system, including the gp91^phox^ and p67^phox^ subunits of NADPH oxide. In the present study, adventitial gene transfection techniques were used to determine the effects of adventitial application of catalase, a crucial anti-oxide enzyme that scavenges H_2_O_2_ *in vivo*, on AngII-induced vascular remodeling. The results of the present study demonstrated that adventitial gene transfection of catalase into carotid arteries significantly attenuated AngII-induced vascular remodeling. In addition, the present study provided further evidence for the participation of vascular adventitia in remodeling and the prominent defensive value of adventitial anti-oxide enzymes. In concurrence with previous studies (29,30), the present study also demonstrated that local perivascular gene transfection targeting the vascular adventitia was a reliable method for the treatment of vascular remodeling in animal models, which may have the potential for use in clinical practice.

Vascular inflammatory responses have traditionally been regarded as ‘inside-out’ processes; however, increasing evidence has supported an ‘outside-in’ hypothesis (31), in which vascular adventitia was considered to be the ‘first-responder’ during the early stages of vascular disease. Several studies have reported a rapid infiltration of leukocytes into the adventitia following vascular injuries (9,32). The present study demonstrated that macrophage infiltration primarily occurred in adventitia following AngII infusion; in addition, following catalase transfection, this AngII-induced adventitial macrophage infiltration was significantly attenuated. This therefore suggested that catalase inhibited AngII-induced vascular wall inflammation. Cascino *et al* (33) showed that adventitia-derived H_2_O_2_ led to vascular relaxation dysfunction and that catalase improved AngII-induced vascular relaxation dysfunction. In concurrence with a previous *ex vivo* study (11), the *in vivo* experiment in the present study confirmed that catalase attenuated adventitial fibroblast phenotypic differentiation and collagen deposition via reducing ROS generation. In addition, the present study demonstrated that the protective mechanisms of catalase proceeded, at least in part, via inhibition of adventitial p38MAPK phosphorylation.

In conclusion, the present study demonstrated that adventitial gene transfection of catalase significantly attenuated AngII-induced vascular remodeling via inhibition of p38MAPK phosphorylation, indicating that adventitia and adventitia-derived ROS had an important role in pathological vascular remodeling.
